# Optoelectronic Tweezers for Single-Cell Research: Principles, Applications, and Prospects‌

**DOI:** 10.34133/cbsystems.0562

**Published:** 2026-06-26

**Authors:** Weiguo Cui, Lu Zhang, Yuguo Dai, Yishen Zhao, Jiayao Zhang, Ao Wang, Xin Pan, Jin Yu, Lingran Kong, Tongtong Li, Lin Feng, Yu Gu, Xue Bai

**Affiliations:** ^1^ School of Biomedical Engineering, Capital Medical University, Beijing 100069, China.; ^2^ Beijing Key Laboratory of Fundamicationental Research on Biomechanics in Clinical Application, Capital Medical University, Beijing 100069, China.; ^3^ School of Mechanical Engineering and Automation, Beihang University, Beijing 100191, China.; ^4^ Department of Bioengineering, School of Engineering, The University of Tokyo, Tokyo 113-8654, Japan.

## Abstract

Single-cell studies offer rich insights into the cellular heterogeneity and function, providing a promising approach for precise diagnostics and personalized treatments. Single-cell research involves initial screening and in-depth analysis. Among available techniques, optoelectronic tweezers (OETs) stand out, offering a noncontact, gentle method that enables flexible, directional control of single cells through dynamic optical patterns. OET is effective in both screening and analysis. This review first introduces the sorting and analysis techniques used in single-cell research and then delves into the principles and development of OET technology, followed by the current achievements of OET in advanced single-cell research. Finally, it discusses and prospects the potential development of OET in the context of single-cell research applications.

## Introduction

Single-cell studies advance transcriptomics, proteomics, metabolomics, and drug evaluation. Due to their complexity, new methods for single-cell research are continuously emerging. Among these, optoelectronic tweezers (OETs) excel at high-throughput performance, contact-free operation, and programmability. OET integrates optical trapping with dielectrophoresis, using projected patterns on a photoconductive layer to generate virtual electrodes, eliminating the need for physical ones. These patterns produce adjustable nonuniform electric fields for precise manipulation of micro- and nanoscale objects. Some reviews on OET and its micro–nano manipulation applications have already been published and are actively utilized as key information sources regarding the technical principles and applications of OET [1,2]. Jorgolli et al. [1] focused on the application prospects of OET in the drug development workflow, including antibody discovery, bioassay development, antibody engineering, and cell line development (CLD). Zhang et al. [2] provided a systematic overview of the principles, chip architecture, and system design of OET, demonstrated the application of the technology in both biological and nonbiological applications, and reviewed the industrialization progress of the technology for the first time. Subsequently, the authors performed a comparative analysis between optical tweezers (OTs) and OET and systematically reviewed their applications in the field of optically driven micromechanics [3].

**Fig. 1.**
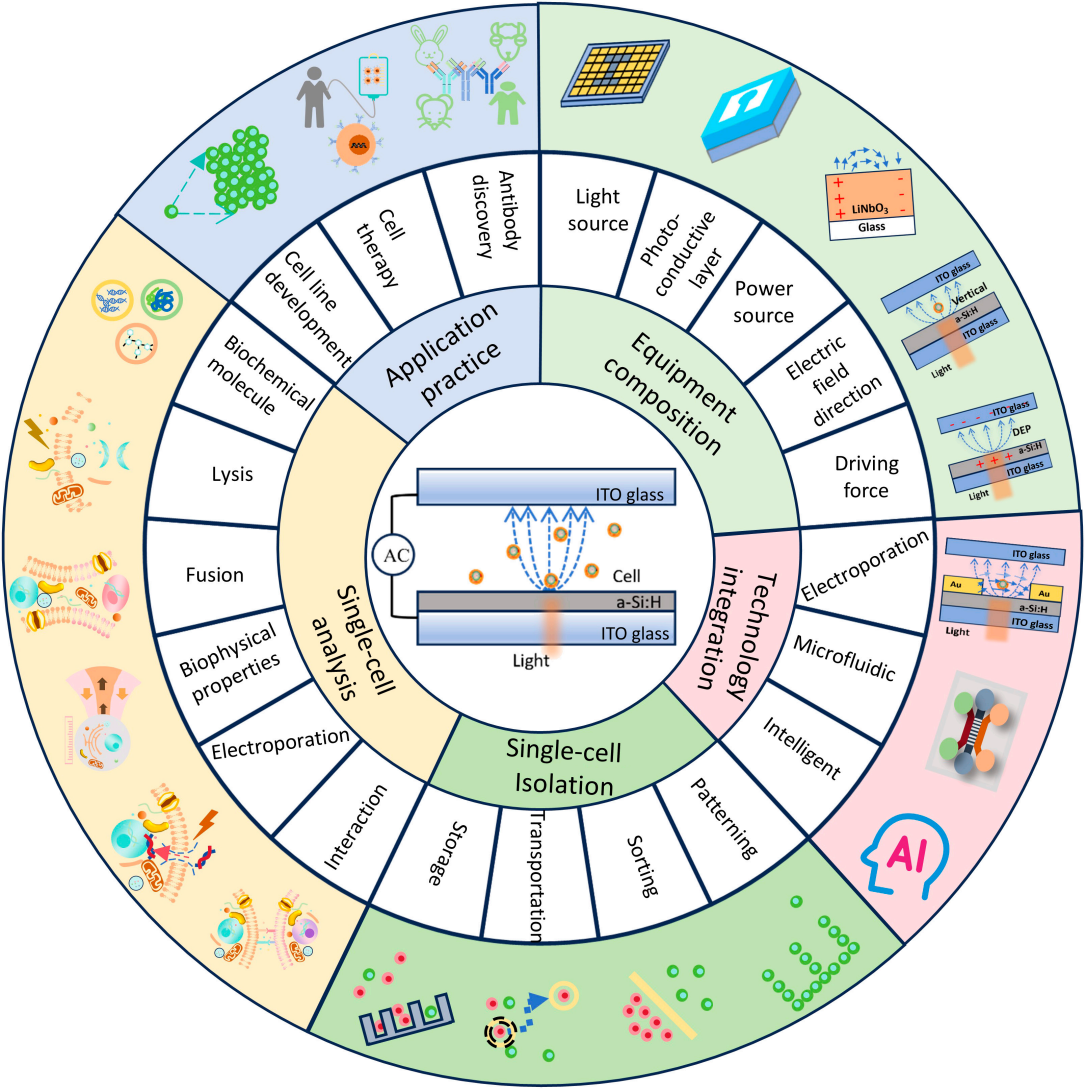
Optoelectronic tweezer technology and its application to single-cell research. The photoelectric tweezers device comprises 5 components: a light source, power supply, photoconductive layer, electric field direction, and driving force. It can be integrated with technologies such as electrolysis, microfluidics, and artificial intelligence. The application of photoelectric tweezers technology in single-cell research encompasses 3 major directions: single-cell manipulation techniques (capture, transport, separation, storage, and patterning), single-cell analysis methods (electrophysiological interaction, biochemical analysis, and spectroscopic analysis), and integrated life science applications (antibody development, cell line screening, and immunotherapy).

In a series of biomedical applications of OET, its role in single-cell research is particularly prominent. This review first provides an overview of the commonly used single-cell research techniques and their corresponding application scenarios in single-cell studies. Then, this review gives shot and clear systematized analysis of the technical principle, driving forces, equipment, and multitechnology integration of OET, influencing their isolation, analysis, and comprehensive application in single-cell research (Fig. 1). The single-cell research was discussed from 2 aspects: initial single-cell screening and subsequent in-depth single-cell analysis. The market-oriented application of OET that integrates virous devices and technologies is listed to provide valuable references for laboratories seeking to enter the market. We aim to provide scholars who are embarking on their initial foray into the domain of single-cell research, as well as researchers who have recently encountered OET equipment, with a relatively comprehensive and lucid understanding of OET technology.

## Single-Cell Research

Single-cell research generally goes through several stages [4,5]: First, cell tissues or cell suspensions are obtained, and then cells are captured and separated from the tissues or mixed cell populations by single-cell sorting techniques. These isolated cells are analyzed for function or lysed for DNA, RNA, or protein analysis [6,7]. The results are then used in applications such as immunoassays and cell therapy. This section describes the common single-cell sorting methods and single-cell analysis methods currently available.

### Single-cell isolation

**Fig. 2.**
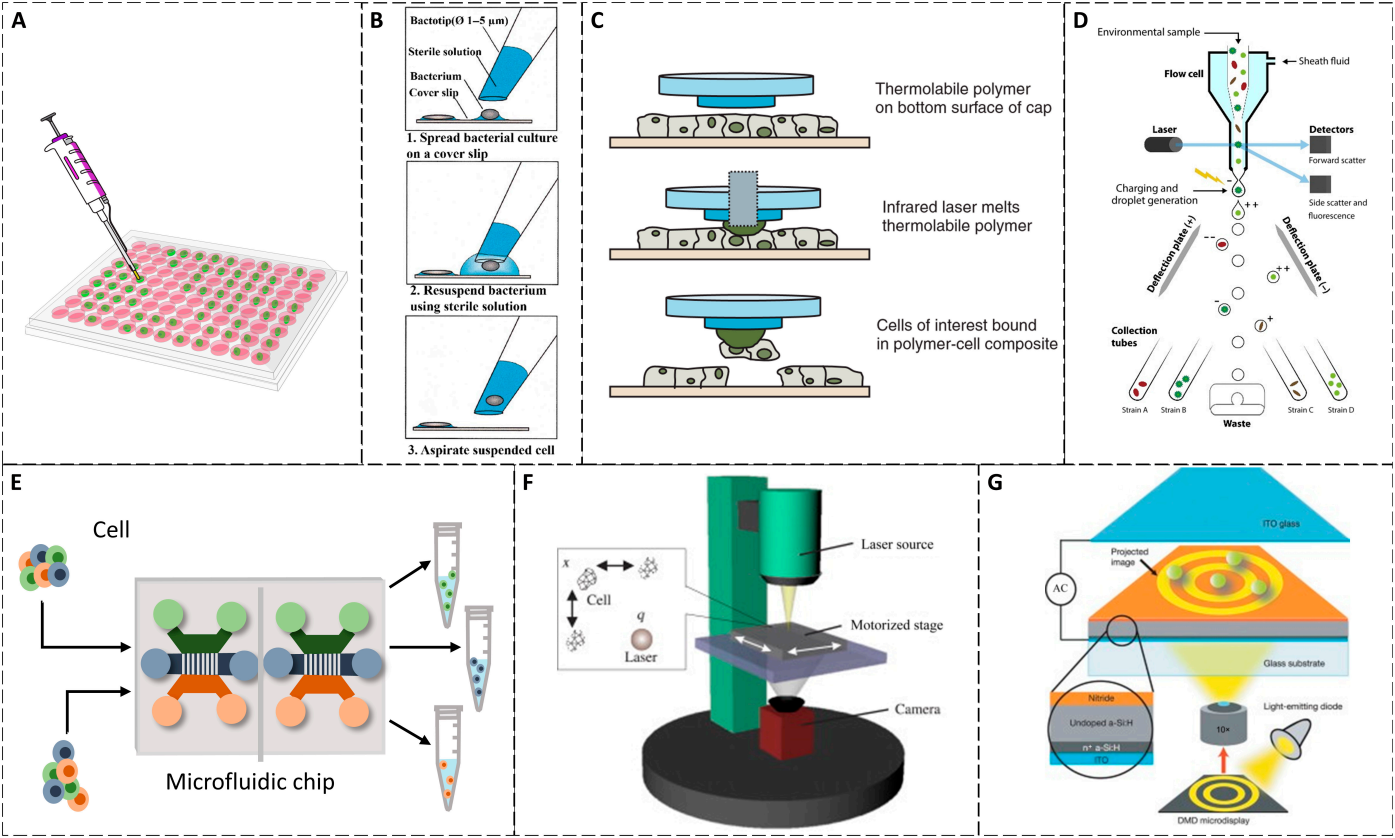
Schematic diagram of single-cell sorting techniques. (A) Limited dilution method. (B) Micromanipulation method. Reproduced from Frohlich and Konig [10]. Copyright 2000, Federation of European Microbiological Societies. (C) Laser microdissection method. Reproduced from Espina et al. [14]. Copyright 2006, Springer Nature. (D) Fluorescence-activated cell sorting. Reproduced from Pereira et al. [15]. Copyright 2018, Elsevier B.V. (E) Microfluidic technique. (F) Optical tweezer technique. Reproduced from Li et al. [34]. Copyright 2013, Elsevier. (G) Optoelectronic tweezer technique. Reproduced from Chiou et al. [36]. Copyright 2005, Macmillan Magazines.

To conduct single-cell studies, the process commences with capturing and isolating individual cells from a tissue or cell population. Initially, this was done using limiting dilution methods [8,9]. Later, researchers developed advanced tools such as micromanipulation [10–13], laser capture microdissection (LCM) [6,14], fluorescence-activated cell sorting (FACS) [7,15–18], microfluidics [19–33], OT [34,35], and OET [2,36] (Fig. 2). Several single-cell manipulation technologies remain in the research stage [37]. Commercializing these technologies requires improving accuracy, ensuring robust performance in complex biological environments, and reducing large-scale implementation costs.

The limiting dilution method is a widely used cloning technique (Fig. 2A). The intended cell line is pipetted from culture wells using either a manual pipette or an automated pipetting system, followed by a cell count [9]. The cell concentration per milliliter is determined and then diluted to ensure an average of one cell per fraction, creating a single-cell suspension. Micromanipulation involves precisely sorting cells under a microscope using tools such as microinjectors and microcapillaries (Fig. 2B) [10–13]. Microscopic manipulation techniques have advanced with objectives that provide long working distances and high magnification. Microorganisms can be observed at 1,000× total magnification using an inverted microscope, while a precise pressure control system enables accurate positioning of capillary tubes for isolating microbial cells. LCM is an advanced technique for identifying cells under a microscope and precisely extracting high-purity cell populations or single cells from tissue sections or live cell cultures using laser technology (Fig. 2C) [6,14]. Researchers examine tissue sections, identify target cells, mark cut areas, excise the tissue, and collect cells using methods such as gravity sedimentation, light pressure rebound, and viscous separation. FACS uses cell size, particle size, and fluorescence to characterize and distinguish cell types in a heterogeneous population [7,15–18]. During sorting, target cells are labeled with fluorescent techniques to differentiate them from the negative control. A high-density stream of suspended cells passes through the laser detection zone in single-file droplets. The fluorescence detection system identifies target cells based on predefined parameters. A piezoelectric crystal encapsulates each cell by generating droplets with varying charges. As these charged droplets pass through an electric field, they are diverted into distinct collection devices, enabling isolation and purification of the target cells (Fig. 2D). Microfluidic chip uses micron-scale channels to manipulate fluids at the nanoliter or picoliter scale. It is widely used for isolating, enriching, and analyzing rare cells with high efficiency and sensitivity [19,20,22–31]. For example, droplet microfluidic technology can generate over 20,000 single-cell droplets per minute [38]. Microfluidic chip can also be combined with detection methods such as fluorescence microscopy and OET for comprehensive analysis of cell morphology and activity (Fig. 2E). OT uses a focused laser beam to capture and manipulate microscopic objects [34,35]. It exerts 2 forces on micrometer-sized particles: the scattering force from photons impact and the gradient force from field intensity variations (Fig. 2F).

OET is a powerful photocurrent technique that uses optically induced dielectrophoresis (ODEP) to control nanoscale and microscale objects [7,12]. Cells in an OET chip are driven by an optical pattern, enabling their capture and separation (Fig. 2G). Each method possesses a unique set of advantages and disadvantages with respect to single-cell sorting and subsequent manipulation capabilities, as well as the scenarios in which these capabilities are applicable (Table 1).

**Table 1.** Comparison of various methods for single-cell manipulation

Technology		Limiting dilution	Micromanipulation	LCM	FACS	Microfluidics	OT	OET
**Advantages**		Easy to operate, low cost, no special equipment required	Visualization, simplicity, low cost	Cells can be isolated directly from tissues	High throughput, multiparameter sorting for suspension cells	Low to high throughput, low sample consumption, highly integrated	Noncontact, undamaged, real-time observation	Higher accuracy, lower cell damage rate, high throughput, flexible maneuverability
**Disadvantages**		Success rate dependent on probability, vulnerable to contamination, labor-dependent	Very low throughput, low level of automation, high operating skill requirements, possible mechanical damage	Low throughput, requires sample fixation, not suitable for live cells, expensive equipment	Dependent on fluorescent labeling, high instrument maintenance costs	Chip channels are prone to clogging, complex design	Low throughput, operational complexity, high cost	Expensive equipment, high system complexity
**Single-cell manipulations**	Encapsulation	√	√	×	√	√	×	√
	Sorting	×	√	√	√	√	√	√
	Trapping	√	√	√	×	√	√	√
	Isolation	√	√	√	√	√	√	√
	Pairing	×	√	×	×	√	√	√
	Patterning	×	√	×	×	√	√	√
	Lysis	×	√	×	×	√	√	√
	Stimulation	×	√	×	×	√	√	√
	Transportation	×	√	×	√	√	√	√
Refs.		[8,9]	[10–13]	[6,14]	[16–18]	[19–32]	[34,35]	[7,12]

LCM, laser capture microdissection; FACS, fluorescence-activated cell sorting.

### Single-cell analysis

Single-cell analysis primarily encompasses examining cellular behavior, genetic profiling, protein characterization, and other biophysical information [32]. Cell behavior analysis primarily focuses on morphology, migration, proliferation, differentiation, and apoptosis. Fluorescence imaging is the most widely adopted technique for observing cellular morphology [39]. Microfluidics [40] and OET [2] platforms serve as versatile analytical tools for investigating cellular value addition, migration, and differentiation. Genetic analysis techniques, mainly for DNA and RNA [41], include sequencing techniques [42,43] such as single-cell RNA/DNA sequencing and DNA methylation sequencing. Protein analysis involves characterizing protein type, quantity, and histological studies [44]. Single-cell proteomics uses mass spectrometry to identify protein markers linked to functional heterogeneity at the single-cell level. Mass spectrometry detects and identifies substances without labeling, enabling precise analysis of proteins, carbohydrates, lipids, and trace elements. Biophysical information analysis evaluates properties such as cell size, density, and dielectric characteristics. Electrochemical detection [45] enables the quantitative analysis of analytes utilizing the electrical signals generated from the electrochemical reaction of the analytes on the electrode surface. Microelectrodes record current responses, characterize cell morphology, and monitor physiological states and biomolecule concentrations. Raman detection [46] is a label-free, noninvasive technique using laser excitation to generate Raman scattering for intracellular molecular vibrational spectra. This enables qualitative and quantitative single-cell analysis and aids in cell sorting. Microfluidics [40,47], OT [48], and OET can be used to manipulate cells for the assessment of their mechanical properties, dielectric characteristics, and size-related information. Flow cytometers can also analyze individual cells, such as cell size, cell volume, cell count, and protein or nucleic acid content. Murphy et al. [49] used a dual-fluorescence flow cytometer to detect the endogenous pH of single cells. Nebe-von-Caron et al. [50] used multicolor fluorescence flow cytometry to achieve functional analysis of bacterial cells at the single-cell level. Newman et al. [51] accomplished single-cell proteomics analysis of acute myeloid leukemia.

## Optoelectronic Tweezers

In 2005, Wu et al. [36] first developed OET, which precisely controls and actuates micro/nanoscale objects by combining optical stimulation with electric fields via semiconductor photoconductivity. OET combines the high resolution of OT with the high throughput of dielectrophoresis, overcoming the limitations of both. It eliminates OT, which suffers from a restricted range and potential cellular damage, and dielectrophoresis demonstrates low flexibility and resolution for individual cell manipulation, although both technologies simplify nonreusable electrode use. The OET device requires only 100,000th of the light intensity needed by OT. It can operate up to 15,000 particle traps in parallel within an area of 1.3 mm × 1.0 mm using incoherent light sources such as light-emitting diodes or halogen lamps. This section will provide an overview of the principles of OET and highlight recent advancements in device development.

### Technical principles

**Fig. 3.**
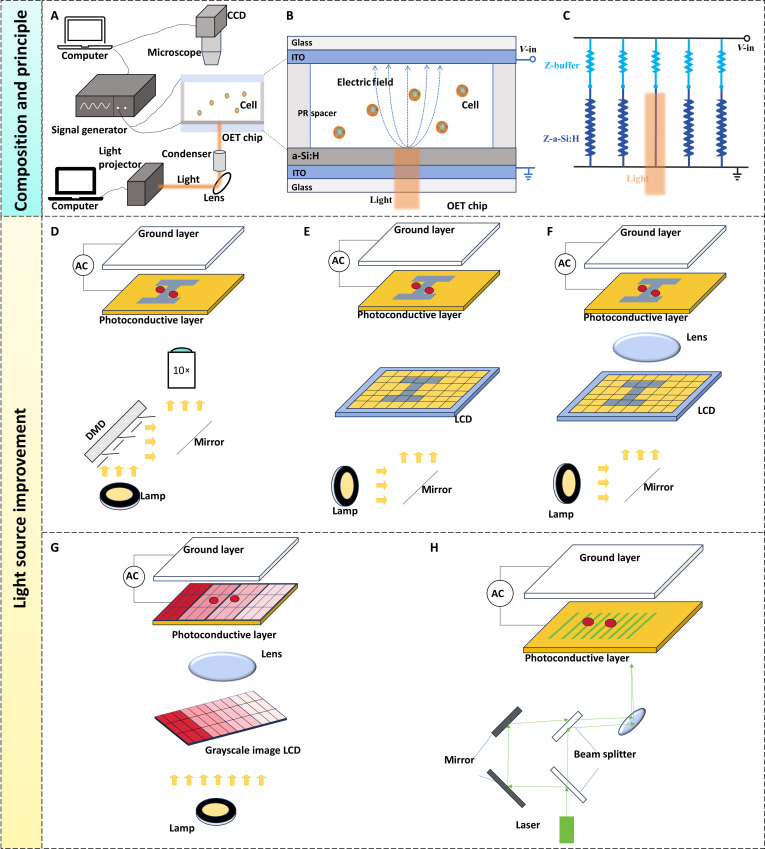
Composition and principle of optoelectronic tweezer (OET) and improvement of light source. (A) Device composition. CCD, charge-coupled device; PR, photoresist. (B) Chip structure. (C) Chip equivalent circuit diagram. (D) Digital micromirror device (DMD) light source. (E) Liquid crystal display (LCD) light source. (F) LCD light source with normal lens. (G) Grayscale LCD image with lens. (H) Interference laser.

A standard OET device consists of power supply, light source, chip, and vision system (Fig. 3A) [52]. The power supply provides the necessary voltage to the chip. Light sources offer adjustable illumination above or below the chip. The camera, integrated with a microscope, captures real-time particle images and sends them to a PC for visualization. The chip is the core component of the OET device, where particle manipulation occurs. Fig. 3B details the architecture of the chip, whose core feature lies in the critical s-Si:H photoconductive layer. The chip consists of 2 indium tin oxide (ITO) conductive glass substrates: The lower substrate is coated with hydrogenated amorphous silicon (a-Si:H), and electrical leads connect to both ITO electrodes. The top and bottom plates are bonded with a spacer to form a sealed chamber for manipulating micro- and nanoscale objects in liquid media. Fig. 3C shows a schematic diagram of the chip’s operating principle. The a-Si:H layer beneath the OET chip is photoconductive. In darkness, its high impedance causes most of the applied voltage to drop across the a-Si:H layer, generating a negligible electric field in the liquid. Under illumination, the layer’s conductivity increases because of electron-hole pair generation, reducing its impedance. This shifts the voltage distribution, applying most of the voltage to the liquid layer and creating a strong nonuniform electric field near the illuminated area. Polarizable particles in the liquid are then manipulated by dielectrophoretic (DEP) force.

### Driving forces

In a standard OET chip, the main manipulating force of the OET device is the DEP force, but in case of varying some parameters (e.g. electric field frequency), AC electroosmosis (ACEO), AC electrothermal (ACET), and electrophoresis (EP) will also play a role [53].

DEP is a phenomenon where polarizable particles experience an electromotive force in a nonuniform electric field. The force intensity depends on the AC field’s frequency and amplitude, the dielectric properties of the medium and particles, and the particles’ shape and size. In OET, when the DEP force is induced by a projected light pattern, it is called ODEP. The main method for calculating DEP forces in OET uses the classical dipole approximation, where the force on a spherical particle is expressed by the following equation:

FDEP=2πr3εmReCM∇E2 (1)

where 𝑟 is the radius of the particle and 𝜀_m_ is the dielectric constant of the medium, *E* is the applied electric field, and [CM] is the real part of the Clausius–Mossotti (CM) factor as described below:

$$\mathrm{CM}=\frac{\varepsilon_{p}^{\prime }-\varepsilon_{m}^{\prime }}{\varepsilon_{p}^{\prime }+2\varepsilon_{m}^{\prime }}$$ (2)

of which $\varepsilon_{p}^{\prime }$ and $\varepsilon_{m}^{\prime }\;$are the complex permittivity of the particle and the medium, where

$$\varepsilon_{p}^{\prime }=\varepsilon_{p}-j\frac{\sigma_{p}}{\omega }$$ (3)

$$\varepsilon_{m}^{\prime }=\varepsilon_{m}-j\frac{\sigma_{m}}{\omega }$$ (4)

Of these, the $\varepsilon_{p}$ and $\varepsilon_{m}$ are the dielectric constants of the particle and the medium, respectively; $\sigma_{p}$ and $\sigma_{m}$ are the conductivity of the particle and medium, respectively; andω is the angular frequency. CM measures polarization rate, determining DEP force polarity. Positive DEP occurs when [CM] > 0, attracting particles to high-field regions such as illuminated areas. Negative DEP happens when [CM] < 0, repelling particles from such regions. Different particle configurations may require model adjustments. For example, in cells with large conductivity and dielectric constant differences between the cytoplasm and membrane, a core–shell model adjustment might be necessary depending on the scenario.

However, strictly speaking, the DEP force in OET chips should be referred to as the ODEP force. In conventional DEP, the electric field distribution is primarily determined by the geometry of the metal electrodes. However, in ODEP, the electric field gradient exhibits strong material property dependence because the photoconductive layer (such as a-Si:H) possesses finite impedance in both dark and illuminated states. Hesselink and colleagues [54,55] have proposed an impedance analysis method based on circuit models. The study indicates that although ODEP exhibits similar mechanical performance to conventional DEP, the ODEP system imposes stricter requirements on the electrical impedance matching (IM) between the suspension medium and the photoconductor. To quantitatively describe this effect, researchers introduced the IM factor. Unlike the CM factor, which characterizes particle polarization properties, the IM factor specifically measures a system’s ability to generate electric field gradients.

For vertical electric field OET devices, the suspension medium and the photoconductive layer are connected in series. Only when the medium’s conductivity $\sigma_{m}$ falls within a specific range, satisfying ${\tilde{Z}}_{p}<{\tilde{Z}}_{m}<{\tilde{Z}}_{d}$, can the system generate an effective electric field gradient and capture force. If the medium is too insulating ($\sigma_{m}$ << $\sigma_{d}$) or too conductive ($\sigma_{m}$ >> $\sigma_{p}$), $f_{\mathrm{IM}}\;$approaches zero, preventing the generation of capture force. $f_{\mathrm{IM}}$ factor is expressed by the following equation.

$$\begin{array}{c} f_{\mathrm{IM}}≜\left|\frac{{\tilde{Z}}_{m}\left({\tilde{Z}}_{d}-{\tilde{Z}}_{p}\right)}{\left({\tilde{Z}}_{p}+{\tilde{Z}}_{m}\right)\left({\tilde{Z}}_{d}+{\tilde{Z}}_{m}\right)}\right| \end{array}$$ (5)

Of these, ${\tilde{Z}}_{m}$ is medium impedance, and the ${\tilde{Z}}_{d}$ and ${\tilde{Z}}_{p}$ are the dark and illuminated photoconductor impedance, respectively.

Further research indicates that lateral electric field OET (LOET) transforms the medium impedance from series to parallel connection. In this mode, LOET eliminates the low-conductivity lower-limit constraint of vertical OET, enabling efficient operation in insulating media (e.g., oil-phase environments) and substantially expanding OET’s medium compatibility. The introduction of the IM factor provides theoretical criteria for optimizing ODEP system design and selecting suitable suspension media. Relevant research content is supplemented in the article.

ACEO flows dominate at relatively low frequencies (below approximately 10 kHz) due to the accumulation of ions at the liquid medium/substrate interface when an alternating electric field is applied, forming an electric double layer that induces ion movement, thereby inducing fluid flow at a slip velocity [56]. ACET flow arises when AC electric fields act on fluids with permittivity/conductivity gradients. In OET, light absorption in the photoconductive layer generates both electron-hole pairs (photoconductivity) and heat via phonons, creating localized temperature differences. These thermal gradients alter the fluid’s dielectric and conductive properties, which, under AC fields, produce volumetric electrothermal forces that drive fluid motion. In typical OET setups with low-power light sources (<100 W/cm^2^), ACET contributes little to the manipulation force [2]. EP [53] is the movement of charged particles toward an opposite electrode under an electric field. This technique separates charged particles moving at different speeds in an electric field. In OET, EP forces need to be considered only when driven by DC or AC signals with frequencies below 10 Hz. For comprehensive theoretical formulations pertaining to ACEO, ACET, and EP, readers are directed to the works in the field [2,53,56].

### Development of OET equipment

Over the past 20 years, the performance of OET devices has substantially improved [2,57,58]. This section will highlight advancements in key components, including the light source, photoconductive layer, power supply, and electric field direction.

#### Light source

The standard light source in OET devices is a digital micromirror device (DMD) (Fig. 3D) [36]. However, DMD projection is complex, which limits portability and increases device complexity. In addition, precise alignment of the lens between the DMD and OET is critical for focusing the image onto the photoconductive layer. Choi et al. [59] reported a portable platform using direct image transfer from a liquid crystal display (LCD) for polystyrene bead manipulation (Fig. 3E). The optical structure in Fig. 3F incorporates an additional conventional lens compared to Fig. 3E. This modification addresses the issue of image blurring on OET devices caused by lens-free methods, which would otherwise compromise the performance of OET systems. The device’s performance was demonstrated through experiments capturing and aggregating red blood cells and screening white blood cells from red blood cells. In addition, a grayscale image with variable intensity values adjusts the electric field strength per pixel, enabling control of *Tetrahymena pyriformis* cells (Fig. 3G) [60]. Yang et al. [61] proposed the laser-induced dielectric EP (LIIDEP) technique. Fig. 3H illustrates its laser configuration, and the feasibility and superiority of the LIIDEP technique were experimentally validated. Compared to projector or laser-based light-induced dielectrophoresis, LIIDEP provides higher resolution, better contrast, stronger force, and precise cell manipulation and facilitates 3-dimensional (3D) assembly of large cell groups.

#### Photoconductive layer

**Fig. 4.**
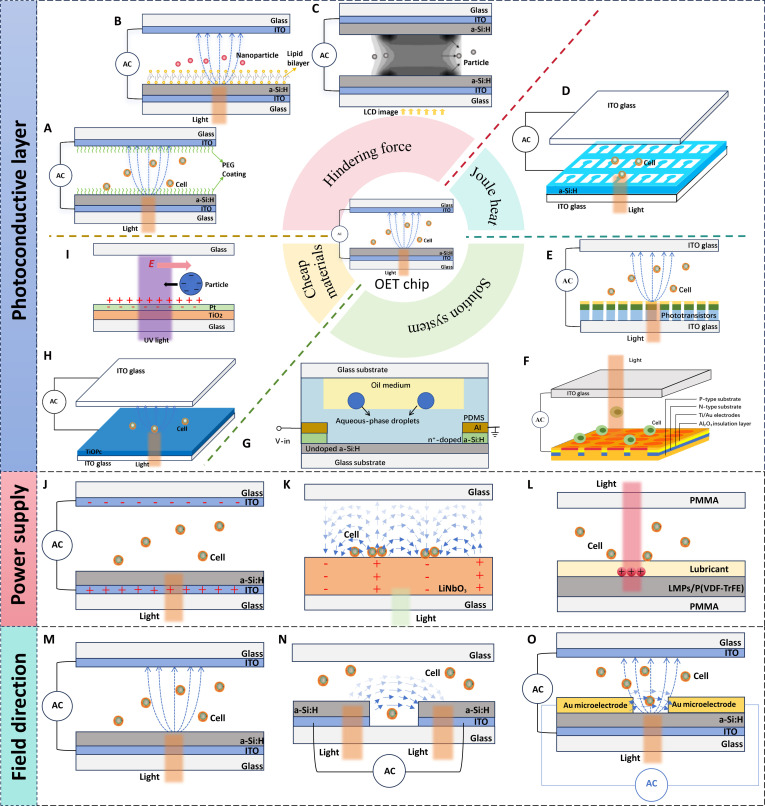
Schematic diagram of improvement in photoconductive layer, power supply, and electric field direction. (A) Polyethylene glycol (PEG) coating. (B) Bilayer lipid coating (C) Upper and lower hydrogenated amorphous silicon (a-Si:H) photoconductive layers (D) Patterned photoconductive layer. (E) Phototransistor photoconductive layer. (F) Self-locking optoelectronic tweezers (OETs). (G) Floating electrode OET. (H) Titanium oxide phthalocyanine (TiOPc) organic photoconductive layer. (I) TiO_2_/Pt membrane. (J) Standard OET using AC power supply. (K) Photovoltaic effect photoconductive layer self-powered. (L) Self-powered thermoelectric conductive layer material using photovoltaic effect. LMPs, liquid metal microparticles. (M) Standard OET generates a vertical electric field. (N) LOET's crossed photoconductive layer generates a lateral electric field. (O) Simultaneous vertical and lateral electric fields.

The photoconductive layer determines OET chip performance and has been optimized for various applications through structural and compositional modifications. OET devices face the challenge of particles migrating and adhering to one side of the surface due to unidirectional forces. Van der Waals forces, electrostatic forces, and nonspecific interactions can hinder precise particle manipulation. Particles attached to the surface may attract others, limiting mobility. For biological samples, interaction with the surface can be fatal. To address this, OET device is modified by replacing the photoelectric layer material to enable 3D manipulation and reduce blocking force through material enhancement. Lau et al. [62] proposed a polyethylene glycol (PEG) coating on OET device to prevent mammalian cell adherence (Fig. 4A). PEG-coated OET increased live cell manipulation efficiency by 30-fold compared to uncoated devices. To address irreversible particle fixation during nanoscale object manipulation, Ota et al. [63] applied a 5-nm lipid bilayer membrane over the optoelectronic conductor substrate (Fig. 4B). Dynamic, reversible, and parallel manipulation of hundreds of gold nanoparticles (<60 nm) in 2D validates device performance. Another solution is 3D manipulation, where particles are suspended in liquid and do not contact the electrode surface. Hwang et al. [64,65] proposed a 3D OET device for microparticle manipulation (Fig. 4C). The platform uses photoconductive layer on the top and bottom substrates. When an AC bias voltage is applied between the layers, projected beams create virtual electrodes on both surfaces, generating an electric field gradient in the liquid. This focuses particles to the center of the chamber through DEP forces from above and below, separating them from the device surface and preventing nonspecific adsorption. This 3D OET achieves higher particle capture efficiency and lower adsorption rates compared to typical OETs.

The photocurrent of OET induces joule heating, causing physiological damage to the trapped cells. Meanwhile, prolonged exposure to light can lead to an increase in cell membrane potential, cell lysis, and even cell death, limiting the application of OET in long-term manipulation of living cells and processing of sensitive samples. Joule heating also increases the evaporation rate of the liquid medium, thereby altering the solute concentration and causing undesirable evaporation-induced fluid shear forces. Zhang et al. [66] proposed a patterned photoconductive layer control scheme. Fig. 4D shows that the device’s middle layer is bare ITO glass, uncoated with a-Si:H. The chip uses potential difference near the ITO and a-Si:H grain boundaries to create a strong electric field gradient above the boundaries. The DEP force repels particles from both internal and external boundaries of the patterned ITO features. This effect persists even after the light is removed. Microbeads remain confined in the OET trap after the projector is switched off. Our group [67] used OET to manipulate Ag-SiO_2_ microspheres as “intermediate particles” to drive neighboring cells or particles through dielectric interaction. This expands the manipulation range 2 to 3 times, increases speed substantially , and maintains high precision. Notably, this method solves cell damage issues from high electric field and light in traditional OET, offering a breakthrough for biological micromanipulation. Meanwhile, the patterned photoconductive layer approach also resolves the issue of resolution limitations in standard OET devices caused by lateral diffusion of photogenerated carriers within the photoconductive layer. The patterned photoconductive layer creates regions of exposed physical electrodes within the device, thereby generating stronger nonuniform electric field gradients. This enables precise confinement of particle capture locations, enhancing the system’s spatial resolution. The Hesselink team [68] proposed a patterned electrode design featuring parallel physical electrode structures on the photoconductive layer to construct electric field components and establish directional force equilibrium points. Under identical spot sizes, the capture region is compressed to 2 distinct positions, substantially suppressing nonspecific background field interference and enabling high-precision separation of micrometer-scale particles.

Illumination also induces a temperature gradient on the surface of the OET substrate. Light absorption within the photoconductive layer transfers its energy not only to electron-hole pairs (i.e., photoconductivity) but also to phonons (i.e., heat). Consequently, the illuminated region may exhibit higher temperatures than the dark region, forming a spatially nonuniform temperature gradient. This temperature gradient drives thermophoresis of particles in the solution, causing them to migrate directionally from the high-temperature zone to the low-temperature zone along the gradient. Wang et al. [69] measured the temperature distribution within the OET chip, confirming the presence of temperature rise and demonstrating that the temperature gradient induced thermophoresis. While temperature gradients may potentially damage biological samples, they can also play a positive role in nonbiological operations, assembly, and manufacturing.

OET devices face inherent limitations in solution-mediated operation. The buffers currently in use are primarily low-conductivity solutions. However, prolonged exposure to these solutions may affect cellular properties such as biochemical composition, morphology, and gene expression. The Bonacci team [70] investigated the effects of buffer systems (low conductivity) on the biochemical, morphological, and mechanical properties of cells during the separation and sorting process using the DEP method. Research findings indicate that in certain buffers, no significant changes were observed in cell viability and growth recovery. In suspension, no noticeable morphological alterations occurred for up to 1 h. Nevertheless, analysis of interleukin-6, inducible nitric oxide synthase, and glyceraldehyde-3-phosphate dehydrogenase markers at the genetic level revealed significant changes in results due to the transfer of cells from minimum essential medium (normal culture medium) to DEP buffer. High-conductivity media distort electric field distribution, reducing manipulation efficiency. Insulating media make it difficult to generate effective DEP forces due to high impedance. Although OET devices using a-Si:H or other organic photoconductors have been demonstrated for many applications, they cannot operate directly with high-conductivity media due to photoconductivity limitations. For example, in an OET chip, most of the voltage drop occurs in the a-Si:H layer when using a high-conductivity cell culture medium, making it difficult to generate the nonuniform electric field required for microscopic manipulation. This is unsuitable for biological applications requiring high-conductivity physiological buffer solutions. To solve this problem, Hsu et al. [71] developed phototransistor-based OET (Ph-OET) devices. Fig. 4E shows a phototransistor fabricated on conductive glass, exhibiting a photoconductivity more than 500 times higher than that of a-Si:H layer. More importantly, their dark-state conductivity is lower than that of the culture medium, while their light-state conductivity is higher (Fig. 4E). Effective capture of live HeLa and Jurkat cells in phosphate-buffered saline and Dulbecco’s modified Eagle’s medium (DMEM) was successfully achieved using Ph-OET. Later, Chiou et al. [72] invented self-locking OETs (SLOTs) based on Ph-OET; SLOT can automatically capture cells in the dark state and release them in the illuminated state (Fig. 4F). It uses a high-density ring array of phototransistor-controlled electrodes for single-cell capture by adjusting electrodes size. When AC voltage is applied to the top and bottom electrodes on the SLOT platform, cells or particles are immediately locked by the nearby ring phototransistor in the dark.

For insulating media, adjusting the electric field strength is difficult because of high impedance. For example, in oil-based systems for 2-phase droplet manipulation, all applied voltages drop across the oil layer even without light. Park et al. [73] reported floating electrode OETs. Fig. 4G shows that the chip features a 0.5-μm nondoped a-Si:H layer on ITO glass, with a 0.1-μm n^+^ a-Si:H layer and a 0.1-μm Al layer at each end. A DC bias voltage applied to the aluminum electrodes generates a uniform transverse electric field across the device, including the oil and silicon layers (Fig. 4G). Local photoconductivity changes, disturbing the previously uniform electric field near the irradiated area. This creates 2 strong electric field regions parallel to the applied field near the edges and a weak field region in the middle, forming an inhomogeneous electric field that penetrates the oil layer. However, the circular beam enables droplet transport only parallel to the applied field, making perpendicular transport difficult. This 1D transport limits floating electrode OET applications in many chemical and biological systems. Park et al. [74] proposed that effective 2D droplet manipulation could be achieved using a double diamond optical light field by improving the optical pattern. This addresses the original equipment’s 1D limitation and enables droplet transport, merging, mixing, parallel processing, and multidroplet operations.

The fabrication of photoconductive layers for standard OET chips must occur in a nitrogen-filled glove box to prevent polymer film damage from water or oxygen. This process, which includes plasma-enhanced chemical vapor deposition, ion implantation, and reactive ion etching, increases complexity. Yang et al. [75] developed OET based on the organic photoconductive material titanium oxide phthalocyanine (TiOPc). This approach is characterized by readily available photoconductive materials, a low-cost optical system, and a user-friendly real-time control interface. TiOPc-based OET substrates are prepared in one process and can be fabricated within 40 min (Fig. 4H). A TiOPc film with a thickness of approximately 200 nm was spin-coated onto ITO glass at a rotational speed of 1,500 rpm for 20 s. After baking at 130 °C for 30 min, the material properties of the TiOPc on ITO glass remained stable for several months under normal operating conditions. Other organic photovoltaic materials, such as bulk heterojunction polymers [76], can be dissolved in organic solvents and applied via spin coating onto ITO substrates to form the photoconductive layer of OET chips. Although organic photoconductive materials are readily available and feature simple processing, issues such as surface roughness currently require resolution. Chen et al. [77] developed an optochemical-electronic tweezer that uses vertical ultraviolet (UV) light to irradiate a TiO_2_/Pt membrane, generating a parallel electric field at the UV boundaries. EP and electroosmosis synergistically move particles (Fig. 4I).

#### Power supply

A standard OET device uses an external AC power supply to provide voltage to the OET chip (Fig. 4J). In contrast, photovoltaic OETs (PVOET) [78–80] operate without an external voltage supply (Fig. 4K). PVOET relies on the bulk photovoltaic effect of noncentrosymmetric materials, where light induces directional carrier transport along the crystal’s polar axis, generating a highly inhomogeneous electric field on the surface that drives PVOET. Zamboni et al. [81] used PVOET to achieve photopolymerization and splitting of confined emulsion droplets in a microfluidic channel. Gao et al. [82] achieved merger, contact, and separation of femtoliter water-containing droplets using surfactants with PVOET, demonstrating cascade chemical microreactivity between microdroplets and particles in an oily medium. Wang et al. [83] developed a thermoelectric photoconductive layer using Ga-In liquid metal microparticle-embedded poly(vinylidene fluoride-*co*-trifluoroethylene) [P(VDF-TrFE)] composites, enabling self-powered OET chips (Fig. 4L). When light is irradiated, the composite material rapidly converts light into local heat, reducing P(VDF-TrFE) polarization and generating surface charge compensation in the irradiated region, creating a local voltage. When light is switched off, the temperature decreases, restoring P(VDF-TrFE) polarization to its initial state and eliminating the surface charge. This OET chip enables manipulation objects of different materials (polymers, inorganics, and metals), phases (bubbles, liquids, and solids), and geometries (spheres, rectangles, and wires), enabling manipulation of biological samples from single cells to cell assemblies, among other functions.

#### Electric field direction

The earliest OET devices generate a vertically orientated electric field within the liquid layer between 2 planar electrodes (Fig. 4M). Since these electrodes must contact the liquid surface, integrating OETs with other devices is challenging. Ohta et al. [84] developed a single-sided OET device with electrodes on the same substrate arranged in a crosswise pattern. The upper pole is plain glass, and the lower pole is ITO glass coated with a-Si:H (Fig. 4N). The device generates an electric field parallel to its plane, known as LOETs. To achieve cell electroporation on an OET device, Valley et al. [85] designed a device generating enabling vertical and lateral electric fields. Two microelectrodes were added, enabling vertical DEP control of cells between the electrodes. A bias voltage applied between the microelectrodes generates lateral DEP forces on particles (Fig. 4O).

### Multitechnology integration with OET

To enhance OET devices capabilities, researchers have optimized their composition and integrated OET with technologies such as cellular electroporation, microfluidics, and intelligent control, substantiallybroadening the application scope.

#### Electroporation and cell lysis

**Fig. 5.**
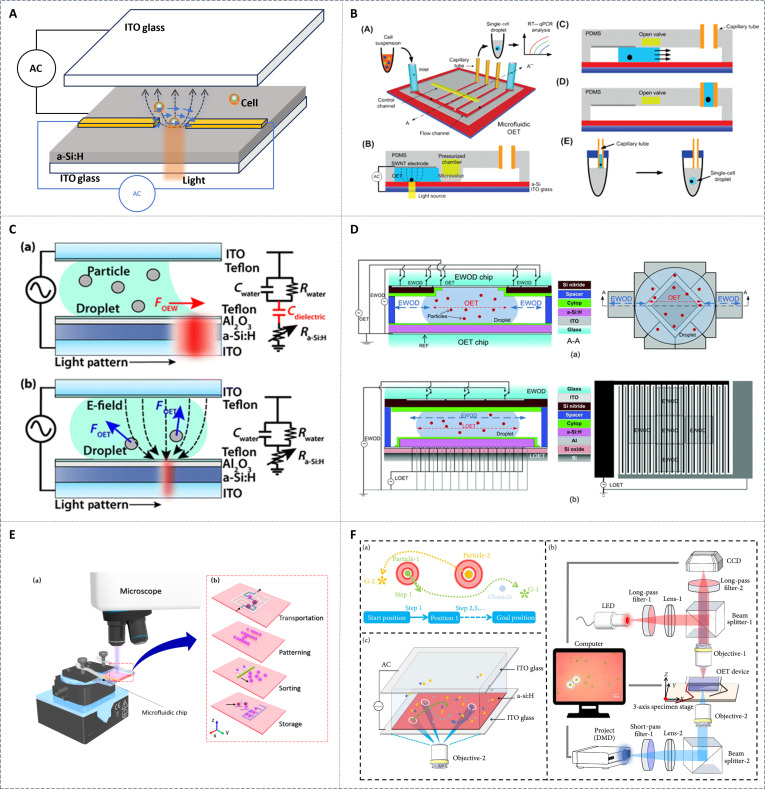
Multitechnology integration of optoelectronic tweezer (OET) devices. (A) Integration of OET and electroporation integration. (B) Integration of OET and microfluidics. RT-qPCR, reverse transcription quantitative polymerase chain reaction; SWNT, single-walled carbon nanotube. Reproduced from Huang et al. [88]. Copyright 2013, Royal Society of Chemistry. (C) Integration of OET and optoelectronic wetting (OEW). Reproduced from Valley et al. [91]. Copyright 2011, Royal Society of Chemistry. (D) Integration of lateral electric field OET (LOET) and electrowetting on dielectrics (EWOD). REF, reference electrode. Reproduced from Shah et al. [93]. Copyright 2009, Royal Society of Chemistry. (E) Multifunctional integrated OET. Reproduced from Liang et al. [96]. Copyright 2022, MDPI, Basel. (F) Intelligent manipulation integration of OET. Reproduced from Liu et al. [94]. Copyright 2022, Beijing Institute of Technology Press.

Combining OET with cell lysis and electroporation technology, a high-throughput and highly parallel platform for precise control of single cells can be constructed. Valley et al. [85] integrated microelectrodes onto the OET chip for cell lysis and electroporation. Fig. 5A shows a pair of gold microelectrodes integrated onto the conductive layer of the chip. Individual HeLa cells were moved to the area between the 2 gold electrodes using OET, and an electroporation bias was applied to achieve cell membrane perforation and lysis.

#### Integration of OET and microfluidics

Microfluidics provides a stable fluid environment, and photonic tweezers achieve dynamic response manipulation. The key to integrating OTs with microfluidic technology lies in the device integration approach. Commonly used methods include the following: (a) Microfluidic channels are fabricated on double-sided adhesive tape and then bonded to the top and bottom plates of the OET chip. Chu et al. [86] fabricated an OET chip with a hollow T-shaped microchannel separated by double-sided tape between the top and bottom layers. Through a 2-step ODEP manipulation, they isolated tumor cells with 81.6% purity from circulating tumor cells; (b) microfluidic channels are prepared on the OET substrate or cover plate using UV-curable polymer SU8 or PEG diacrylate (PEGDA) and then the cover plate or substrate is bonded with epoxy resin.Our group [87] integrated OET with a microwell array microfluidic chip (SU8 on bottom plate) for single-cell capture, sorting, and light-guided retrieval based on DEP. The single-cell capture rate exceeds 91.9%; (c) by integrating gold mesh or carbon nanotubes with polydimethylsiloxane (PDMS), a silicon-based organic polymer, photovoltaic conversion devices with PDMS microfluidic channels were fabricated. This integrated approach simultaneously forms the OET top plate and spacer layer. Chiou et al. [88] integrated OET with a multilayer microfluidics by embedding single-walled carbon nanotube electrodes in a PDMS structure (Fig. 5B). This platform isolates target cells based on their optical characteristics (e.g., size, shape, and fluorescence signal) and transports them to individually isolated chambers at a speed of up to 10 μm/s. The integration of OET and microfluidic technology also enables other cellular control functions. Hsiao et al. [89] used ODEP combined with optically induced, locally enhanced electric fields in a microfluidic device to achieve precise and automated cell pairing and fusion. Chou et al. [90] designed a 4-stage cell separation structure based on an optical virtual cell filter within a microfluidic system. This approach combines differential flow rates and varying DEP forces to achieve high-purity cell separation. The integration of OET technology with microfluidics represents a critical pathway for advancing from “single-cell manipulation” to “fully integrated lab-on-a-chip” systems, holding significant research potential.

#### Integration of OET and OEW

The optoelectronic wetting (OEW) effect is used as simultaneous droplet control and micro-object manipulation [91]. Photovoltaic wetting uses light-induced virtual electrodes to selectively convert hydrophobic surfaces to hydrophilic ones, attracting nearby microdroplets. Fig. 5C details the architecture of the chip, whose core feature lies in the droplet being sandwiched between the top ITO electrode (coated with polytetrafluoroethylene) and the bottom ITO electrode (coated with a-Si:H, Al_2_O_3_, and polytetrafluoroethylene layers). According to the OET device principle, applying an external bias between 2 ITO electrodes creates an electric field that exists predominantly in the high-resistance a-Si:H layer in the absence of light. Upon light irradiation, the conductivity of the a-Si:H layer increases dramatically, reducing across the dielectric and liquid layers. If a large portion of the field is in the liquid layer, the electric field gradient will exert a DEP on the particles within the droplet. If the field is primarily in the dielectric layer, the droplet will experience a net organic electrical force toward the irradiated region. When the externally applied electric frequency is below a critical frequency, the capacitor’s impedance dominates the liquid layer impedance, enabling OEW operation. Above the critical frequency, the dielectric impedance becomes negligible compared to the liquid resistance, transitioning the device from OEW to OET and enabling droplet and particle manipulation switching. Valley et al. [91] combined OET and OEW technologies to encapsulate single and multiple HeLa cells within 75-nl droplets. The Yu team[92] used TiOPc as the photoconductive layer, with microchannels formed via UV polymerization of PEGDA material. Integrating OEW technology with PEGDA microfluidic fabrication enabled droplet generation and manipulation between ITO glass chips. As an emerging droplet control technique, OEW combined with OET will enable broader functional applications.

#### Integration of LOET and EWOD

In single-cell research, besides manipulating individual cells, it is frequently essential to expose them to diverse environmental conditions and stimuli, such as pharmacological or mechanical interventions. Droplets serve as cellular microchambers, providing a controlled microenvironment for studying isolated cells. OET enables manipulation of individual particles under continuous microfluidics, but isolating them into discrete droplets remains challenging. Whereas electrowetting on dielectrics (EWOD) enables precise cutting, merging, and control of droplets. By integrating EWOD with OET, the aforementioned challenges can be effectively addressed. The Shah team [93] attempted to combine a standard OET device with EWOD in the first-generation device but found it challenging to demonstrate the complete sequence of EWOD–OET operations due to the mutual exclusivity of their operating regions. They then integrated EWOD and LOET in a second-generation device. The 2 LOET electrodes, located on the same substrate, overlaps with the EWOD actuation electrodes, resolving the issues of the first-generation device (Fig. 5D). The OET chip handles high and low voltages, so the EWOD chip no longer needs to accommodate OET. The OET’s ability to penetrate the EWOD region has successfully enabled cell control and droplet segmentation for sequence manipulation.

#### Intelligent integrated control system

OET can control and fabricate micro- and nanomaterials, but its devices are complex and require professional operation. Researchers have enhanced the intelligence and functionality of the OET system [94,95]. Our group [96] designed an OET system for multifunctional operation. The system uses standard 4× to 60× microscope heads and can manipulate particles of various sizes (2 to 150 μm), including both living and nonliving objects (Fig. 5E). The system integrates optical micropatterns for capturing, storing, parallel transporting, and patterning particles, enabling user-friendly operation. In addition, temperature measurements under different objective lenses confirm that the system does not generate excessive heat to destroy biological particles. Liu et al. [94] proposed a discretized operation method with centralized decoupled path planning for transporting particles on an OET platform (Fig. 5F). An approach using the Kuhn–Munkres algorithm assigned target particles to target locations. With visual feedback, a partially observable Markov decision process-based path planning method dynamically determined motion strategies to avoid collisions. Experiments transporting multiple particles (polystyrene microspheres and 3T3 cells) with different morphologies confirmed the effectiveness of the proposed OET-based manipulation method for flexible and efficient manipulation tasks.

## Applications in Single-Cell Research

When the Wu team invented the OET device, they conducted experiments to demonstrate its ability to manipulate cells and particles [97,98]. They used dynamic image-driven control to collect particles, sort microbeads of different particle sizes through a comb structure, and aggregate live red and white blood cells. Over 20 years, OET devices have been developed for applications including manipulation, lysis, counting, sorting, DNA molecule manipulation, carbon nanotube capture, and semiconductor and metal nanowires separation [2]. These manipulation capabilities highlight the potential of OET devices for applications in both biological and nonbiological fields. This section summarizes the application of OET devices in single-cell research.

### Single-cell isolation manipulations

The isolation and manipulation of single cells are fundamental applications of OET equipment. Single-cell isolation requires recognizing, capturing, controlling movement, and compartmentalizing, which are interconnected processes. OET supports cell identification, separation encapsulation [87,97], and patterning. Cell identification, separation, and encapsulation aid in CLD and heterogeneity analysis, while cell patterning facilitates intercellular communication, organoid formation, and cell interactions.

#### Cell identification, isolation, and encapsulation

**Fig. 6.**
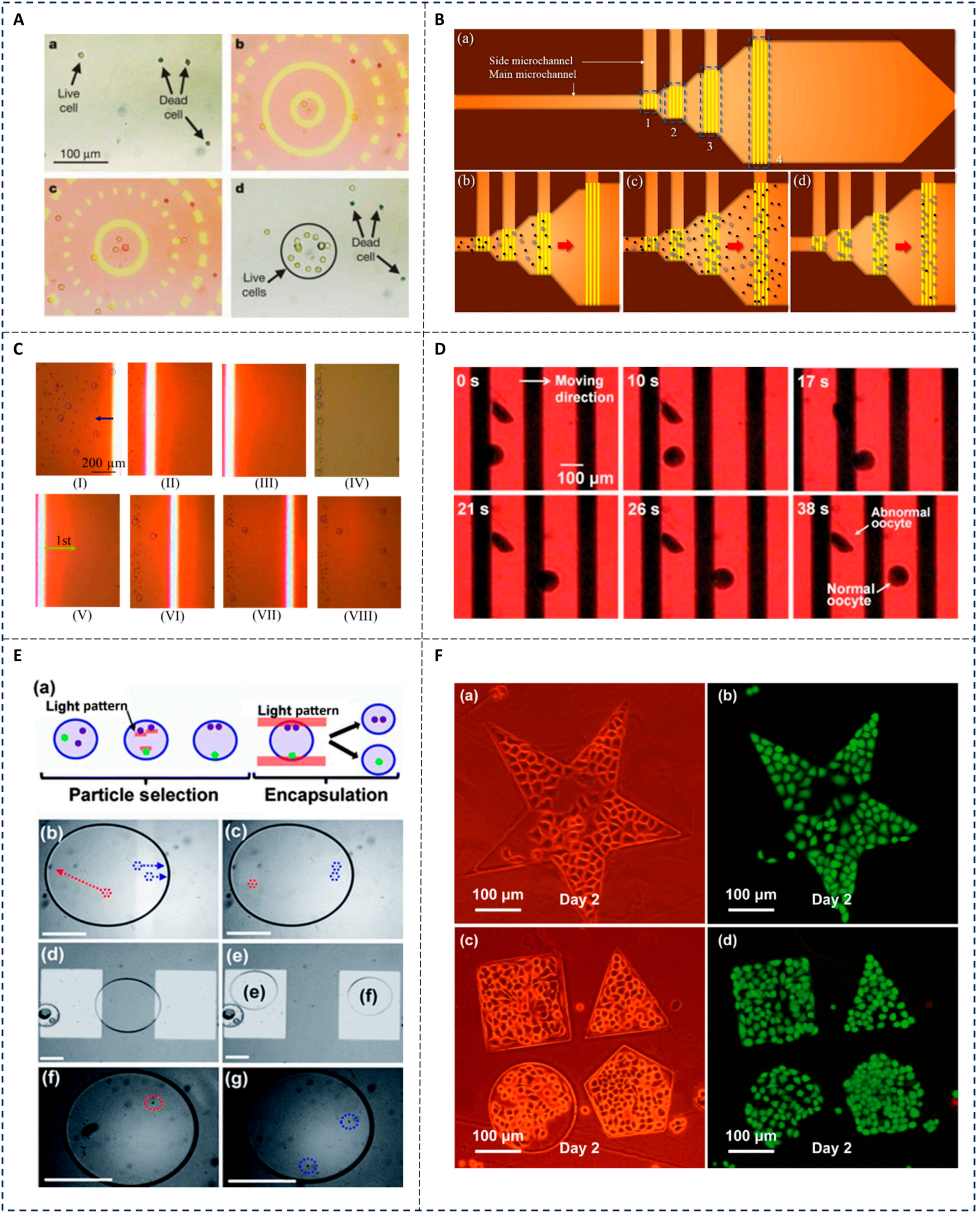
Application of optoelectronic tweezer (OET) technology in single-cell sorting. (A) Concentration of B cells in live dead cells. Reproduced from Chiou et al. [36]. Copyright 2005, Macmillan Magazines. (B) Light-based 4-stage cell separation structure. Reproduced from Chou et al. [90]. Copyright 2017, Elsevier B.V. (C) Moving light bar to selectively separate cancer cells from leukocytes. Reproduced from Huang et al. [101]. Copyright 2012, Elsevier B.V. (D) Screening of normal and starved abnormal oocytes. Reproduced from Hwang et al. [102]. Copyright 2009, AIP Publishing. (E) Encapsulation of HeLa cells in microdroplets. Reproduced from Valley et al. [91]. Copyright 2011, Royal Society of Chemistry. (F) Cell patterning. Reproduced from Liu et al. [103]. Copyright 2014, Royal Society of Chemistry.

In an OET chip, cells experience forces such as DEP, gravity, and friction. These forces vary on the basis of factors such as cell size, dielectric constant, gravity differences, and optical pattern size, enabling OET to identify, manipulate, and separate cells. For living cells, their membranes are selectively permeable, maintaining an ion concentration difference between intracellular and extracellular environments. In contrast, dead cells in low ionic media experience ion diffusion, which dilutes intracellular ions. These differences in dielectric properties cause live cells to experience positive DEP forces and dead cells experiencing negative DEP forces in OET chips. As a result, live cells are attracted to the illuminated region and collected at the center of the contracted halo pattern, while the dead cells remain uncollected. Wu et al. [36] exploited to selectively concentrate human B cells from a mixture of living and dead cells (Fig. 6A). Garcia et al. [99,100] used the OET device to achieve highly sensitive screening of live and dead sperm, quantify OET forces for cell identification, evaluation, and sorting, and ensure no DNA damage during manipulation, improving the success rate of intracytoplasmic monosperm injections.

In the OET chip, the DEP force on the cell is also influenced by the light pattern thickness. For mixtures of cells with different sizes, such as a mixture of Jurkat and HeLa cells, Ohta et al. [84] used varying DEP force magnitudes with differently coarse scanning rays to sort the cells in the OET device. The OET from the thinner guidewire is strong enough to hold Jurkat cells during scanning but not strong enough for HeLa cells. Instead, HeLa cells are transported by the force from the thicker tailwire, enabling the separation of cells of different sizes. Chou et al. [90] designed a 4-stage cell separation structure using optical virtual cell filters in a microfluidic system to exploit the size difference between leukocytes and circulating tumor cells (Fig. 6B). By combining flow rate and DEP force differences, cells are selectively attracted to the light bar array, achieving high-purity cell separation. Results showed that this method isolated cancer cells with 94.9% ± 0.3% and 54% ± 7% recovery.

Huang et al. [101] used a moving light bar with varying speeds to selectively separate PC-3 cancer cells from leukocyte (Fig. 6C). Hwang et al. [102] identified and screened normal and starved oocytes using the speed difference between gravity and antigravity modes of the OET device, caused by the dielectric property difference between normal and starved abnormal oocytes (Fig. 6D). This method can develop automated, interactive tools for selecting fertilizable oocytes. At the same time, the combination of OET and OEW technologies (see the “Integration of OET and OEW” section) enables the realization of cell encapsulation functionality. Valley et al. [91] has combined OET with OEW to enable the encapsulation of single and multiple HeLa cells in 75-nl droplets (Fig. 6E).

#### Cell patterning

Cell patterning technology enables precise control of cell adhesion, shape, and size, playing an important role in cell biology, tissue engineering, and high-throughput drug screening. Liu et al. [103] used the OET device to manipulate MCF-7 cells into triangular-, square-, and star-shaped patterns and confirmed their activity for 48 h (Fig. 6F). Chiou et al. [97] demonstrates the manipulation capabilities of the OET device by forming patterns with blood cells and B cells in low conductivity solutions. Yang et al. [75] demonstrated microscopic images of human liver cells patterned into snowflake shapes within seconds using programmable light patterns in the OET system. Cell patterning technology enables the study of cellular interactions, including adhesion, migration, and communication. By precisely controlling cell arrangement, it can simulate natural tissue structure and function, supporting tissue engineering development. Patterning cells onto specific regions allows for easy observation and analysis of drug responses, accelerating promising drug candidates screening. Designing substrates with specific physicochemical parameters (e.g., morphology, roughness, and temperature) induces selective cell adhesion, mimicking in vivo cell–microenvironment interactions.

### Single-cell analysis

#### Cell–cell interactions

**Fig. 7.**
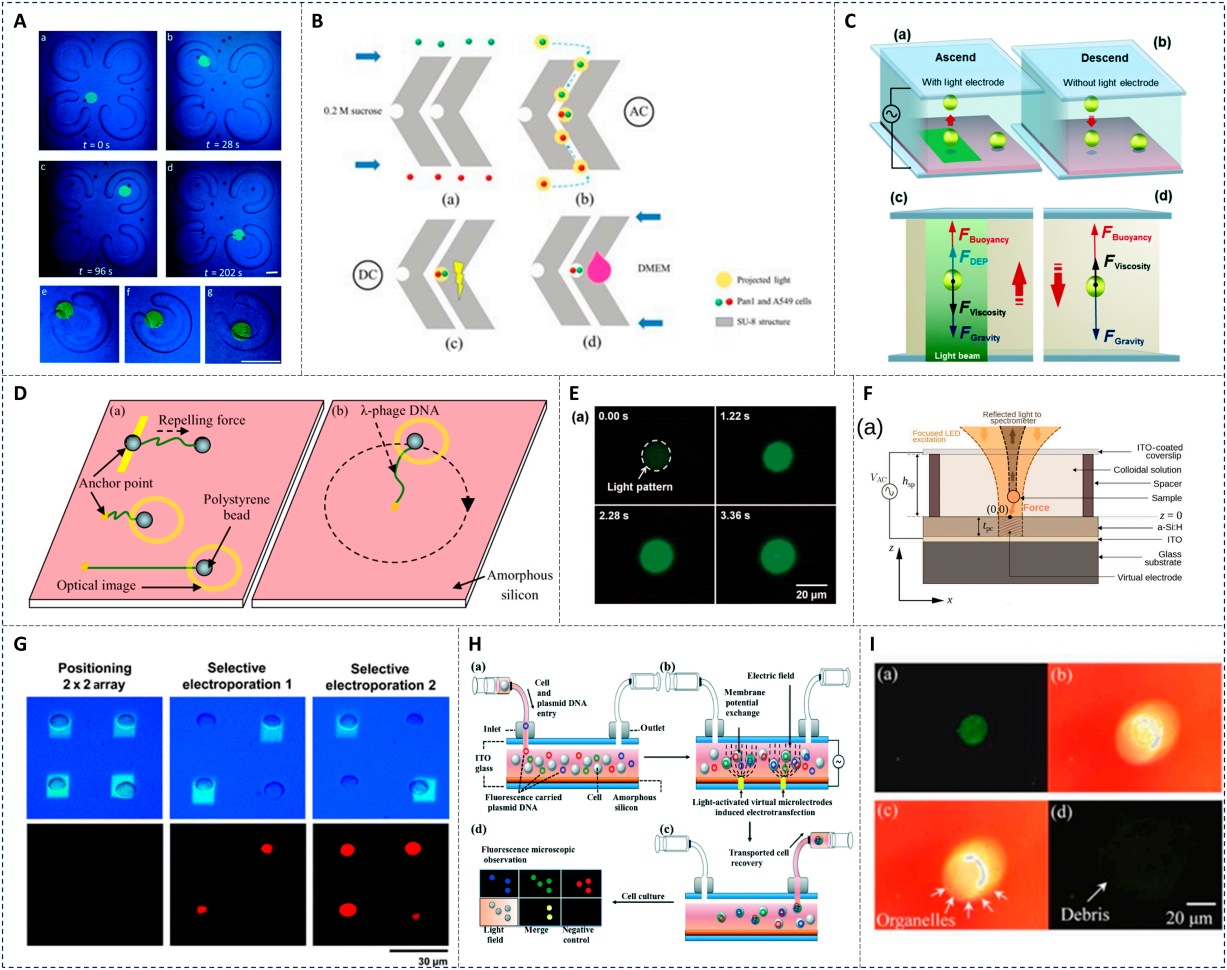
Application of optoelectronic tweezer (OET) technology to single-cell analysis. (A) Cell-to-cell interactions. Reproduced from Ke et al. [104]. Copyright 2017, Royal Society of Chemistry. (B) Cell fusion. Reproduced from Hsiao et al. [89]. Copyright 2018, AIP Publishing. (C) Physical property measurements. Reproduced from Zhao et al. [105]. Copyright 2014, Royal Society of Chemistry. (D) Controlled microbead stretching to move DNA. Reproduced from Lin et al. [107]. Copyright 2009, OPTICA Publishing Group. (E) Controlled dextran aggregation concentration. Reproduced from Hwang et al. [108]. Copyright 2009, American Chemical Society. (F) Spectral analysis. Reproduced from Zaman et al. [110]. Copyright 2024, AIP Publishing. (G) Cell electroporation. Reproduced from Valley et al. [111]. Copyright 2009, Royal Society of Chemistry. (H) Combination of OET and microfluidics for electroporation and gene transfection. Reproduced from Wang et al. [112]. Copyright 2014, Royal Society of Chemistry. (I) Cell lysis. Reproduced from Lin and Lee [113]. Copyright 2009, AIP Publishing.

Analysis of natural killer (NK) cell activity is widely used to study interactions between immune cells and target cells. Ke et al. [104] used TiOPc-based OET to enable direct cell–cell contact for studying immune cell interactions and analyzing NK cell behavior in real time (Fig. 7A). NK cells and target cells were placed in PEGDA hydrogel microwells to ensure direct contact, facilitating observation of their interaction. It is noteworthy that the TiOPc photoconductive layer material used in this technology is precisely the photoconductive layer material proposed by researchers in the “Photoconductive layer” section. This material preparation process offers advantages such as simplicity and efficiency, readily available raw materials, and rapid synthesis speed.

#### Cell fusion

Hsiao et al. [89] performs accurate and automated cell pairing and fusion on a microfluidic device using ODEP and locally enhanced electric fields. After pairing cells with OET and transporting them to the receiving site, the projected light pattern generates a locally enhanced electric field, inducing an appropriate transmembrane potential in the contact region and achieving a 9.67% fusion rate between Pan1 and A549 cells (Fig. 7B).

#### Measurement of cell physical properties

Cells in the same cycle phase have similar volumes, but differences in mass and density reveal their physiological stats. Zhao et al. [105] reports a new technique for rapid measurement of single-cell density and mass on an optically induced electrokinetic microfluidic platform, combining sedimentation theory, computer vision, and particle manipulation (Fig. 7C). A time-controlled pattern of projected light irradiated a selected area on the optically induced electrokinetic microfluidic chip containing cells. The cells were lifted to a specific height above the chip surface and then allowed to free-fallen back. Computer vision algorithms accurately tracked cell movement. By correlating trajectories with sedimentation theory, the volume, mass, and density of each cell were quickly determined.

OET can calculate the capacitance and conductance of cell membranes, key parameters for assessing cellular phenotypes and status. Specific membrane capacitance serves as a marker-free biomarker to determine cell development and differentiation and distinguish normal from cancer cells. Liang et al. [106] observed and tracked the trajectories and textures of Raji cells, MCF-7 cells, human embryonic kidney 293 cells, and K562 cells using a microscopic motion tracking technique. Cross-spectral analysis and curve fitting were then used to determine cell membrane capacitance and conductance. Liu et al. [52] developed an OET system for automatic characterization of individual cell membrane capacitance. The system uses a computer vision tracking algorithm to control the sweep range, measure cell crossing frequency and radius, and calculate the specific membrane capacitance.

#### Biochemical molecular manipulation analysis

OET can directly control biochemical molecules such as DNA, proteins, and polysaccharides. Chiou et al. [56] concentrated λ-phage DNA molecules into the illuminated area using blue fluorescence excitation light patterned with partially closed iris rings on an inverted microscope. Lin et al. [107] bind the ends of individual DNA molecules to microbeads, which are manipulated via optical image interactions to indirectly control the DNA, enabling both its extension and rotation (Fig. 7D). Hwang and Park [108,109] integrated OET with fluorescence microscopy to control polysaccharides, proteins, and fluorophores, enabling modulation and detection of local chemical concentrations and measurement of diffusion coefficients for various glucose (Fig. 7E).

#### Spectral analysis

Zaman et al. [110] has proposed a spectroscopic tweezers using a single focused beam as both the OET beam and the spectroscopy detection beam (Fig. 7F). A focused broadband beam manipulates and isolates individual trace samples, while reflected light from the sample is analyzed to obtain characteristic spectra for label-free spectral characterization. This study conducted verification experiments on yeast cells, showing the potential of spectral tweezers as a readily achievable analytical tool.

#### Cell electroporation, lysis and gene transfection

OET devices can achieve electroporation by varying device parameters or combining OET with microelectrode electroporation. When the bias voltage is adjusted, the electric field strength increases. If it exceeds the threshold, cell membrane perforation occurs. Valley et al. [111] used standard OET to find that the optimal electric field required for electroporation of HeLa cells was in the range of 1.4 to 2.3 kV/cm. Cells could be manipulated or electroporated in parallel by a simple change in the device bias (Fig. 7G). As mentioned in the “Electroporation and cell lysis” section, researchers can combine OET technology with electroporation to achieve cell membrane perforation or lysis. The Valley team [85] has integrated microelectrodes onto OET chips for cell lysis and electroporation. HeLa cells were moved between 2 gold electrodes using the OET device, and cell membrane perforation was achieved by applying an electroporation bias. Wang et al. [112] established a microfluidic-assisted electroporation technique by combining OET and microfluidics with light-activated virtual. As shown in Fig. 7H, electroporation and transfection were achieved using low voltage and AC, efficiently delivering single or triple DNA fluorescent plasmids into 15 types of mammalian cells and successfully expressing fluorescent proteins.

Cell lysis is a key technology for extraction proteins and nucleic acids, widely used in research. However, existing methods face significant challenge: Chemical lysis cannot target specific cells; bubble-rupture-generated pressure wave is difficult to control; and microfluidic devices, while capable of lysis cells, struggle to target specific cells within a group. This issue can be resolved using the LOET device mentioned in the “Electric field direction” section, which is achieved by altering the direction of the electric field. Valley et al. [85] performed in situ lysis of individual cells along the LOET device using a standard data projector in parallel. Reducing the applied bias voltage enables DEP operation on cells without lysis. Lin and Lee [113] reported selectively lysing individual cells in a group by adjusting OET parameters, spot diameter, and light intensity. In addition, the method enables selective disruption of the cell membrane without damaging the nucleus (Fig. 7I).

### The market-oriented applications of OET in single-cell research

After over 20 years of development, researchers have leveraged OET in single-cell research to achieve significant results across various fields, including antibody discovery, CLD, tumor immunology, and cellular therapeutics. Commercially mature OET equipment such as the Beacon single-cell photoconductive platform has extensively integrated microfluidic technology (see the “Integration of OET and microfluidics” section), EWOD technology (see the “Integration of LOET and EWOD” section), and intelligent integrated control systems (see the “Intelligent integrated control system” section), thereby expanding the functionality and controllability of OTs technology. These advances offer unprecedented opportunities for cutting-edge scientific research and accelerated bioproduct development (Table 2).

**Table 2.** The latest commercial applications of the OET platform

Application	Article	Function	Time
Antibody discovery	Protective antibodies target cryptic epitope unmasked by cleavage of malaria sporozoite protein [127].	An antigen-agnostic antibody discovery workflow has been developed using the Beacon® system.	2025
	Transcobalamin receptor antibodies in autoimmune vitamin B12 central deficiency [128].	Beacon system analyzes tens of thousands of single B cells for antigen-specific antibody secretion.	2024
	Alpaca single B cell interrogation and heavy-chain-only antibody discovery on an optofluidic platform [114].	Screening of alpaca variable domain of the heavy chain of heavy-chain antibodies using the Beacon platform.	2023
Cell line development	SiMPl-GS: Advancing cell line development via synthetic selection marker for next-generation biopharmaceutical production [129].	The Beacon system facilitated the development of the SiMPl-GS selection system based on the AND gate principle for cell line generation.	2024
	Combined approach of selective and accelerated cloning for microfluidic-chip-based system increases clone specific productivity [130].	The Beacon platform enables the rapid generation of high-yield Chinese hamster ovary cell clones.	2024
	Preclinical development of a molecular-clamp-stabilized subunit vaccine for severe acute respiratory syndrome coronavirus 2 [131].	Beacon cloning screening facilitates the development of a severe acute respiratory syndrome coronavirus 2 vaccine.	2021
Cell therapy	Phase I/II study of adaptive manufactured lentiviral anti-CD20/anti-CD19 chimeric antigen receptor T cells for relapsed, refractory mantle cell lymphoma [132].	Beacon platform’s “High-Precision” chimeric antigen receptor T cell functional characterization data.	2024
	The screening, identification, design, and clinical application of tumor-specific neoantigens for TCR-T cells [133].	The Beacon® platform cultivates and identifies functional T cells, followed by T cell receptor sequencing.	2023
Tumor immunity	Utilizing immunogenomic approaches to prioritize targetable neoantigens for personalized cancer immunotherapy [121].	Characterize and isolate individual cells exhibiting the desired phenotype and then proceed to transcriptomic analysis or off-chip amplification.	2023

#### Antibody discovery

**Fig. 8.**
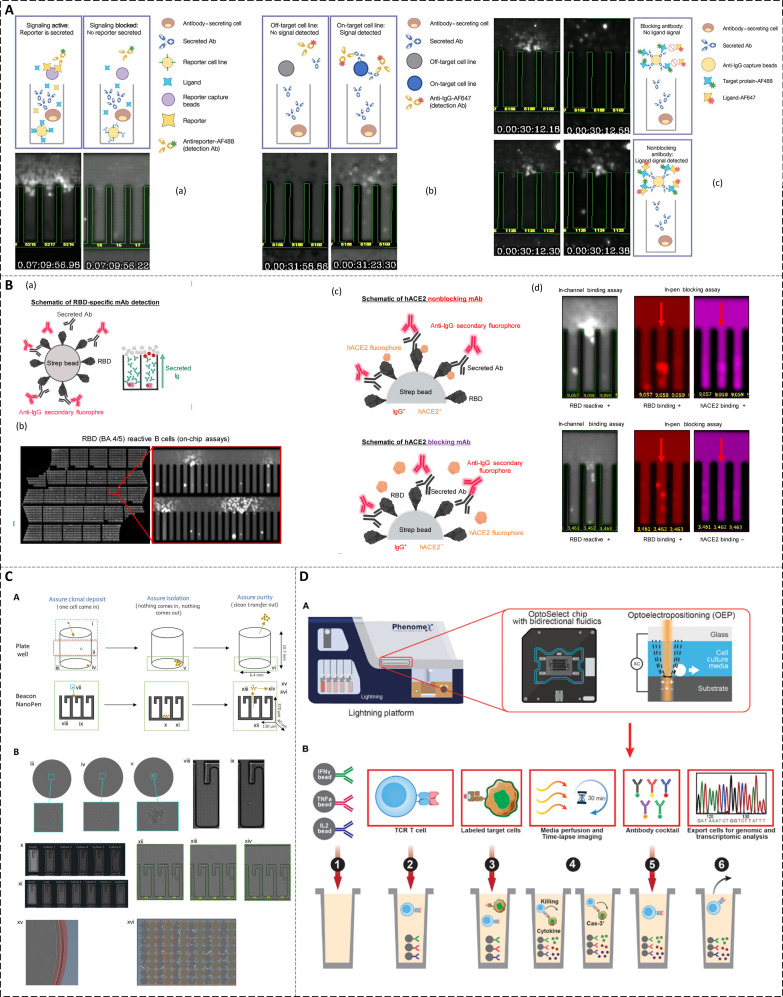
Antibody development and cellular immunotherapy using the optoelectronic tweezer (OET) device. (A) Determination of biologically relevant properties of antibodies (Ab) secreted by individual B cells using the OET platform. IgG, immunoglobulin G. Reproduced from Shapiro et al. [114]. Copyright 2023, Oxford University Press. (B) Functional assays from individual antigen-reactive B cells. mAb, monoclonal antibody; hACE2, human angiotensin-converting enzyme 2. Reproduced from Yu et al. [118]. Copyright 2024, Frontiers in Immunology. (C) Comparison of cell line development using subcloning and OETs. Reproduced from Le et al. [120]. Copyright 2020, John Wiley and Sons. (D) Workflow of cellular immunoassay performed on the photoconductive platform. IFNγ, interferon-γ; TNFa, tumor necrosis factor a; IL2, interleukin-2; TCR, T cell receptor. Reproduced from Shah et al. [121]. Copyright 2023, Frontiers in Immunology.

In recent years, multispecies antibodies for human, mouse, rabbit, and alpaca have been developed using the Beacon single-cell photoconductive platform [114–118]. Shapiro et al. [114] developed a method to enrich, activate, and culture B cells from alpacas in vitro. The activated cells (more than 10,000 per chip) were introduced into a photoconductive platform, where antigen–antibody binding assays and 2 reporter cell- and microbead-based blocking assays were conducted to screen for target antibodies. Eligible positive cells were then exported for downstream sequencing and analysis (Fig. 8A). As shown in Fig. 8B, Yu et al. [118] used the photoconductive platform to isolate monoclonal antibodies recognizing the S-protein receptor-binding domain (RBD) from individuals with severe acute respiratory syndrome coronavirus 2 Omicron infection and identified 2 candidate, 2173-A6 and 3462-A4. Both exhibited strong RBD binding and effectively neutralization a wide range of Omicron pseudoviruses. The Beacon platform enables rapid isolation and characterization of potentially neutralizing antibodies from humans within a 10-d time frame.

#### Cell line development

CLD is a complex but very critical process in the development of biological drugs. Recent studies [119] indicate that although various companies have implemented diverse CLD platforms, the Beacon platform remains the most widely adopted technical approach for single-cell cloning, as reported by 38% of respondents during the CLD experiments phase. The first stage of CLD involves loading cells from the transfected library into the OptoSelect chip’s liquid flow channels. The imaging software then identifies individual cells, applies optoelectric positioning to target them, and moves each cell into a NanoPen chamber. The chip was perfused with culture solution to maintain viability for several days of online culture. As cells expanded in the chamber, fluorescent signals were assayed at multiple time points to identify clones with desired properties. After 4 to 6 days of online incubation, clones with desired characteristics were selected from the chip for recovery. Optoelectric positioning exports a single clone (8 to 20 cells) from its NanoPen chamber to the flow channel. The system flushes the clone off the chip into individual wells of a 96-well plate for expansion using a culture solution. Le et al. [120] validated the workflow using Chinese hamster ovary-derived cell lines expressing green or red fluorescent proteins, confirming >99% monoclonality in the photoconductive platform system (Fig. 8C).

#### Cell therapy and tumor immunity

The Beacon single-cell photoconductive system has achieved significant results in an increasing number of new applications for T cell single-cell functional characterization. The platform enables cytotoxicity assays at single-cell resolution, dynamically tracking cytotoxicity kinetics and revealing diverse killing behaviors. It also supports multiple cytokine secretion analyses to identify key cytokines driving cellular function and uses fluorescence to detect specific surface marker expression on T cells or target cells. Shah et al. [121] detail the workflow for T cell functional analysis in single-cell photoconductive systems (Fig. 8D). This capability enhances understanding of neoantigen-reactive cells in cancer immunotherapy, reveals cancer-immune cell interactions and influencing factors, and accelerates the development of cell-based immunotherapies.

## Summary and Perspectives

By integrating various biochemical analysis and detection technologies, OET has become a powerful platform for single-cell cloning, functional analysis, and analysis of genomic, transcriptomic, and proteomic heterogeneity. It will be widely applied in antibody discovery, CLD, and cell therapy, supporting the advancement of personalized therapies.‌

Although OET technology has demonstrated numerous mature applications, the high cost of commercial OET equipment currently limits its widespread adoption in ordinary universities or enterprises. The photoconductive layer widely used in current OET chips remains a-Si:H, whose fabrication process is complex and costly. While alternative materials such as organic photoconductive compounds (TiOPc) are now available—offering readily accessible materials and short spin-coating preparation times with simple operation—issues such as surface roughness still require resolution. The search for novel photoconductive materials, novel phototransistor structures, and more advanced fabrication techniques remains an urgent priority in OET research. Given the relatively high cost of commercialized OET equipment, research into compact, intelligent, and cost-effective devices is essential.

Currently, when using OET equipment, raw samples require pretreatment before injection into the device. To facilitate rapid adoption by researchers, particularly those in interdisciplinary fields, improvements are needed in the OET device’s intelligence, integration, and standardized operating procedures. For instance, diverse biological samples (such as tissue digestion fluids, sputum, and blood clots) require specific pretreatment based on sample type (such as dilution, tissue digestion, and centrifugation enrichment) to meet OET processing requirements. Therefore, OET devices could integrate preprocessing modules. Users would simply insert sample tubes and select the corresponding preset preprocessing workflow option. The instrument would then automatically perform the preprocessing before injecting the sample into the core chip area for manipulation. Integrated preprocessing module technology is already widely used in various analytical devices, such as high-throughput gene sequencers and automated liquid chromatography–mass spectrometry systems. It enables automated whole blood separation, sample dilution, protein precipitation, solid-phase extraction, and other functions. Therefore, leveraging related technologies, a preprocessing module system tailored for OET devices can be developed. Naturally, integrating OET devices with downstream analysis modules such as omics analysis represents another significant research direction. By combining microporous arrays or droplet microfluidics, OET enables the precise delivery of cells into microchambers containing barcoded magnetic beads, achieving an integrated “manipulation–capture–library construction” workflow. Users can select and combine modules according to their specific research needs, which will facilitate the widespread adoption of OET devices.

Standardization poses a barrier to the widespread adoption of OET. DEP force is highly dependent on medium conductivity, and there is currently a lack of standardized buffer formulations for different cell types. Simultaneously, inconsistencies in voltage, frequency, and light intensity standards across laboratories make it difficult to reuse experimental parameters across platforms. Professional personnel are required to adjust the equipment. Therefore, it is essential to establish quality control standards for the performance of photoconductive materials and other equipment parameters. Currently, OET applications are limited to in vitro chips, with in vivo applications posing significant challenges. OET requires a 3-layer structure (top electrode, interlayer solution, and bottom photoconductive layer), making it nearly impossible to construct this sandwich-like electric field environment within living organisms. However, as a tool, OET enables in vitro functional screening, cultivation, and detection of cells obtained from living organisms, thereby facilitating antibody development and immunotherapy.

OET requires cultivating individual cells and labeling target cells for single-cell analysis—a time-consuming, labor-intensive, and costly process. Deep learning now allows scientists to identify and track cells accurately and efficiently in large image datasets without labeling [122,123]. These systems have been used to classify circulating tumor cells by morphology [124]. Future applications of lightweight models on OET may facilitate classification based on more complex functionalities rather than simple morphological features. This function allows OET to screen functional cells such as B cells or chimeric antigen receptor T cells using white light images and monitor stem cell status and differentiation in real time without fluorescence labeling.

Fabricating micro–nano structures on the photoconductive layer increases OET chip production costs. Array OT technology enables mask-free, reconfigurable particle manipulation through dynamic holographic optical fields, eliminating the need for such structures [125]. Integrating an OT array into an OET device requires addressing light and electric field distortions from overlapping optical paths and localized heating caused by high-power lasers and current flow in the photoconductive layer. Synchronous interference between the 2 optical paths can be reduced using artificial-intelligence-optimized spatial coordination or temporal activation alternation of the laser array and electrode field. Using particles [67] or microgears [126] as intermediaries in OET to manipulate single cells reduces localized heating from direct manipulation.

In summary, this review provides a comprehensive overview of the use of OET devices in the field of single-cell research in terms of the principles and development of the devices as well as their proven applications. It also serves as a preliminary introduction for scholars who are new to the field of single-cell research and the study of OET devices.

## Data Availability

Data sharing is not applicable to this article, as no new data were created or analyzed in this study. All data presented in this review were reused under permission.
